# Interpreting Risk and Impact Measures in Nursing Research: Implications for Evidence-based Practice

**DOI:** 10.17533/udea.iee.v44n1e14

**Published:** 2026-03-31

**Authors:** R. Mauricio Barría P.

**Affiliations:** 1 RN, MSc. Clinical Epidemiology, DrPH. Institute of Nursing, Faculty of Medicine, Universidad Austral de Chile. email: rbarria@uach.cl https://orcid.org/0000-0002-3764-5254 Universidad Austral de Chile Institute of Nursing Faculty of Medicine Universidad Austral de Chile Chile rbarria@uach.cl

**Keywords:** nursing research, data interpretation, statistical, measures of association, measures of association, exposure, risk or outcome, nursing, practical, evidence-based practice., investigación en enfermería, interpretación estadística de datos, medidas de asociación, medidas de asociación, exposición, riesgo o desenlace, enfermería práctica, práctica clínica basada en la evidencia., pesquisa em enfermagem, interpretação estatística de dados, medidas de associação, medidas de associação, exposição, risco ou desfecho, enfermagem prática, prática clínica baseada em evidências.

## Abstract

**Objective.:**

To analyze the conceptual foundations, calculation, and interpretation of key risk and impact measures used in nursing research, emphasizing their relevance for evidence-based clinical practice.

**Content synthesis.:**

This article reviews measures of risk or association, including relative risk, odds ratio, and hazard ratio, as well as absolute impact measures, such as risk difference, absolute and relative risk reduction, absolute risk increase, and numbers needed to treat and harm. Using examples from recent primary studies conducted in clinically relevant nursing contexts, the manuscript illustrates step-by-step calculations and interpretations, highlighting the complementary roles of relative and absolute measures in clinical decision-making.

**Conclusion.:**

An integrated understanding of risk and impact measures is essential for critical appraisal of nursing research and to assess the real clinical relevance of interventions. The combined use of these measures supports more informed, safe, and context-sensitive nursing care decisions, reinforcing evidence-based nursing practice.

## Resumen

**Objetivo.:**

Analizar los fundamentos conceptuales, el cálculo y la interpretación de las principales medidas de riesgo y de impacto utilizadas en la investigación en enfermería, destacando su utilidad para la práctica clínica basada en evidencia.

**Síntesis del contenido.:**

Se revisan las medidas de riesgo o asociación, como el riesgo relativo, *odds ratio* y *hazard ratio*, así como las medidas de impacto absoluto, incluyendo la diferencia de riesgos, la reducción y aumento absoluto del riesgo, la reducción relativa del riesgo y los números necesarios a tratar y dañar. Mediante ejemplos provenientes de estudios primarios recientes en contextos clínicos relevantes para la enfermería, se ilustra el cálculo paso a paso de estas medidas y su correcta interpretación, enfatizando las diferencias entre efectos relativos y absolutos y su complementariedad en la toma de decisiones clínicas.

**Conclusión.:**

La comprensión integrada de las medidas de riesgo y de impacto es esencial para una adecuada lectura crítica de la literatura científica y para valorar la relevancia clínica real de las intervenciones de enfermería. El uso conjunto de estas medidas permite apoyar decisiones de cuidado más informadas, seguras y contextualizadas, fortaleciendo la práctica de enfermería basada en evidencia.

## Resumo

**Objetivo:**

Analisar os fundamentos conceituais, o cálculo e a interpretação das principais medidas de risco e de impacto utilizadas na pesquisa em enfermagem, destacando sua utilidade para a prática clínica baseada em evidências.

**Síntese do conteúdo:**

São revisadas as medidas de risco ou associação, como risco relativo, *odds ratio* e *hazard ratio*, bem como as medidas de impacto absoluto, incluindo diferença de riscos, redução e aumento absoluto do risco, redução relativa do risco e números necessários para tratar e para causar dano. A partir de exemplos de estudos primários recentes em contextos clínicos relevantes para a enfermagem, são apresentados os cálculos e a interpretação dessas medidas, enfatizando a complementaridade entre medidas relativas e absolutas na tomada de decisão clínica.

**Conclusão:**

A compreensão integrada das medidas de risco e de impacto é fundamental para a leitura crítica da literatura científica e para a avaliação da relevância clínica das intervenções em enfermagem. O uso conjunto dessas medidas contribui para decisões de cuidado mais informadas, seguras e contextualizadas, fortalecendo a prática baseada em evidências.

## Introduction

Research in nursing practice is fundamental to offer evidence-based quality care in search of providing safe and efficient care, and with such, achieve better health results of patients and users in different contexts in which the nursing practice is carried out. In keeping with the nature of the different performance settings in which nursing provides care, research also covers a wide range of topics, from the development of new approaches in clinical care to the study of health interventions and policies. Consequently, through research, nursing professionals can examine and assess care practices and identify improvement areas to develop new strategies to address healthcare challenges. Notwithstanding, this requires sufficient skills to review and critically evaluate published studies, considering their validity, relevance, and applicability in specific clinical situations. 

Research interpretation is a key tool to move towards a safer, more effective nursing profession committed to continuous improvement. Within the context of evidence-based practice (EBP), this ability permits transforming scientific knowledge into specific clinical actions that impact positively on patient health. According to Melnyk and Fineout-Overholt,^1^ health professionals must be capable of locating, analyzing, evaluating, and using relevant evidence to guarantee that patients have the certainty and conviction that their professionals base their care on evidence to obtain optimal results. Thus, to effectively apply EBP, Polit and Beck^2^ indicate that nurses must be capable of critically analyzing and interpreting research results, considering aspects like the study design, sample size, internal and external validity, and clinical significance of the findings. This implies not only understanding the statistical methods, but also evaluating if the results are applicable to their specific clinical environment. In this sense, it has been described that nurses throughout the world are familiarized with EBP, have positive attitudes towards it, and believe in its worth to improve the quality of care and patient results; nevertheless, they perceive that their own knowledge and skills are insufficient to make use of it and do not employ the best evidence in the practice.^3^

The competence or ability to analyze research and determine the level of empirical evidence is essential to address continuous change in the nursing clinical practice. Under these circumstances, it is recognized that nurses’ lack of research knowledge and skills due to insufficient and non-standardized nursing curricula at different levels of training, including undergraduate education can no longer be a barrier associated with problems that limit the use of EBP.^4^ Moreover, it has been found that EBP is associated with nurses using evidence in the practice and the principal role they perform, given that it is observed that head nurses, administrative nurses, and educators are more familiar with EBP than the clinical nurses.^3^

Quantitative studies use statistics to report their findings. Upon analyzing this research and then evaluating the validity of the study results, it is necessary to examine precisely those numerical results. Hence, health professionals planning to use the results from quantitative studies need overall understanding of how to interpret the results that are precisely reported in that section of each article.^1^ In a prior article^5^, I discussed critically the distinction between statistical significance and clinical significance, emphasizing that results with statistically significant p values may lack practical importance, while clinically relevant effects may not reach statistical significance in studies with low power. Said work proposed the need to incorporate measures that allow estimating the magnitude of the effect and its usefulness for patients, health professionals, and health systems. Within this scenario, nurses are required to differentiate and comprehend the nature and significance of different measures employed as indicators of risk, association, or impact and which constitute key elements when evaluating the results of a given research and its usefulness for the practice. Further, and in a purely practical context, in many healthcare centers the quality and patient safety units or departments use daily measures that describe relevant facts, such as tracers and sentinel indicators that allow tracking the frequency of these cases and looking for possible causes or associated factors. Additionally, this constitutes an element that translates into quality of care.

In line with this proposal, this article seeks to develop an applied methodological approach to interpret risk and impact measures, using examples extracted from recent nursing articles. The aim is to provide practical tools that permit translating the results from quantitative research into clinically actionable information, thus strengthening EBP.

### Risk and impact measures in clinical research

For adequate interpretation of evidence, it is necessary to distinguish among risk measures, which describe the strength of association among groups, and impact measures, which quantify the absolute effect of an intervention and its clinical relevance. Risk measures help determine whether an intervention is effective, while impact measures help to decide if it is worth implementing. From this EBP perspective, it should also be mentioned that no single risk measure is sufficient on its own. Relative measures permit identifying associations, while absolute and impact measures are indispensable to decide if an intervention should be implemented, prioritized, or scaled in the actual clinical practice.

Definitions and calculations for the distinct association, risk, and impact measures have been broadly described in standard reference texts for epidemiological training and for EBP.^1^^,^^6^^-^^9^ From there, to try to explain the application of the main risk and impact measures within the limited space of an article, I will use recently published nursing works, highlighting key aspects to exemplify.

### Risk measures (association): conceptual foundations and interpretation

Absolute risk (AR) is the likelihood that an event occurs in a specific group during a defined period. It indicates the real frequency of the event in a population. It is the base to calculate clinical impact measures given that it corresponds conceptually to the cumulative incidence of an event (for example, illness, death, recovery) when longitudinal follow-up is available. It should not be confused with the incidence rate, nor can it be estimated in cross-sectional or case-control studies.

For its calculation, use:




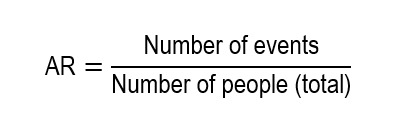




Relative risk (RR) is an association measure that expresses the ratio between the probability of an event occurring in an exposed or intervened group and the probability of occurrence of the same event in a reference group (control). This indicates the probability (that is, the risk) that the result occurs in a group compared with the other. The group with the particular condition or intervention of interest tends to be the study focus. The RR is defined as the ratio between two risks so that when it comes to a longitudinal study - randomized controlled trial (RCT) or cohort-, these risks correspond to the cumulative incidence of the event in each group. Its estimation is obtained from:




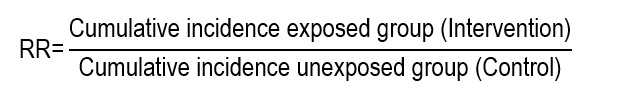




The result of the calculation indicates that, if the RR is < 1 there is risk reduction with the intervention, while an RR > 1 suggests increased risk. Besides its report in individual studies, the RR is useful to compare the direction and strength of the effect among studies, especially in RCT and meta-analysis. Although this measure is widely used in these studies, its clinical interpretation is limited when presented in isolated manner because it does not provide information on the absolute frequency of the event or on the number of patients directly benefitting from the intervention. 

The odds ratio (OR) is an association measure that expresses the ratio between the odds of occurrence of an event in the exposed group and the odds of the same event in the unexposed group. The odds correspond to the ratio between the probability of an event occurring and the probability that it does not occur; therefore, the OR compares said ratio between two groups. It is used mainly when it is not possible to estimate risks or direct incidences. It constitutes the characteristic association measure of case and control studies and of logistic regression models. Although it is often interpreted as an approximation of the RR, this equivalence is only valid when the event of interest is infrequent. In common outcomes, the OR tends to deviate from the RR and can exaggerate the magnitude of the effect, so its clinical interpretation requires special caution in nursing.

To calculate the OR, apply: 




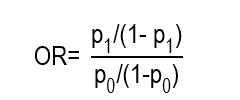




p₁ = probability of the event in the exposed group

p₀ = probability of the event in the unexposed group

As well as the interpretation of the RR, when the OR is equal to 1 there is no association, when it is > 1 there is a greater odds of the event in the exposed group and when it is < 1 there is a lower odds of the event in the exposed group.

The hazard ratio (HR) is an association measure that compares the instantaneous rate of occurrence of an event between two groups over time and it is typical of survival studies. Through the instantaneous risk quotient, it expresses how many times, at each instant within the follow-up, the event is more or less likely in one group compared to another, considering the time until the occurrence of the event. The HR describes a relative effect over time, but does not report directly about the cumulative risk or about the absolute impact. Due to this, its clinical interpretation must be complemented with survival curves or estimates of cumulative risks at specific times. Similar to the interpretation of the RR and OR, when the HR is equal to 1 there is lack of risk (no association), when it is > 1 risk increases, and when it is < 1 there is lower risk.

**Chart 1:**
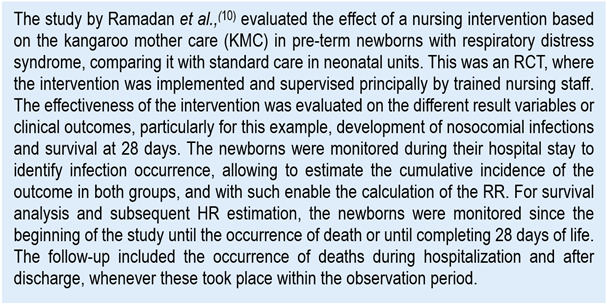
Example of clinical scenario to evaluate RR and HR

**Chart 2:**
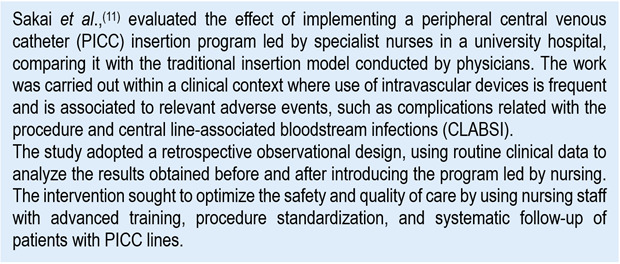
Example of clinical scenario to evaluate OR and HR

For didactic purposes, we will take as reference two recently published articles (Chart 1 and Chart 2) that allow recognizing the reported risk measures, as well as exemplifying the calculation, interpretation, and its implications for nursing practice.

The study by Ramadan *et al*.,^10^ compared the incidence of nosocomial infections among pre-term children who received KMC implemented by nursing and those who received standard care (AR per group). These incidences were 15% and 33.3%, respectively. The result reported is an RR = 0.45 (CI_95%_: 0.27-0.75; *p*<0.001). Applying the previously described equation:




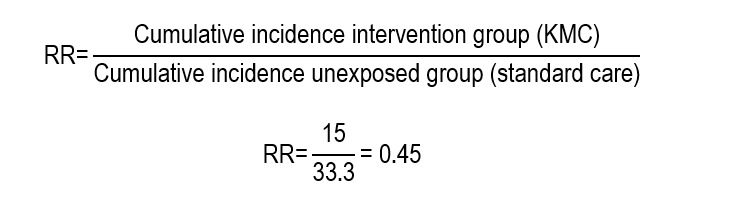




This means that, given the RR value < 1, the pre-term infant from the KMC group had lower risk of developing infection. Specifically, the neonates from the KMC group had 45% risk of developing nosocomial infections compared with those from the control group, which indicates a significant risk reduction with the nursing intervention. In other words, the probability of nosocomial infection in the KMC group was 55% lower than in the control group.

Further, applying a Cox proportional hazards statistical model, the researchers found that the KMC intervention implemented by nursing was associated with a significant survival improvement at 28 days, expressed as: adjusted HR = 0.42 (CI_95%_: 0.28-0.63; *p*<0.001). An HR < 1 indicates that the neonates from the KMC group had a lower instantaneous rate of death than those from the control group. Specifically, the HR of 0.42 indicates that, over 28 days of follow up, the neonates who received KMC had approximately 58% less instantaneous risk of dying compared with the neonates who received standard care. This was calculated considering:




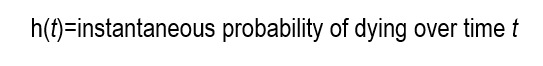




The HR compares those functions between groups:




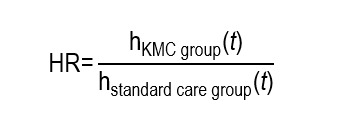




As a clarifying note, it is important to point out that when reporting an adjusted HR (or any other adjusted measure), the comparison between groups is not done in crude manner but rather considering that newborns are not identical at the start of the follow-up. Within this context, the analysis recognizes that some infants may have a higher baseline risk of mortality due to their initial clinical characteristics, independent of the nursing intervention. The statistical model controls these differences, so that the HR reflects the association between the intervention -for example, the KMC- and neonatal mortality beyond said baseline characteristics. In the study analyzed, the fit variables included the gestational age, weight at birth, and the initial clinical severity, measured through the Silverman Anderson Score.

Studies, such as that herein analyzed, show that the same nursing intervention can generate evidence expressed through distinct hazard measures, each one responding to specific clinical questions. Integrated comprehension of these measures results fundamental to interpret adequately the results and support decision making in EBP.

In the study by Sakai *et al*.,^11^, selection of risk measures was determined by the nature of the outcomes analyzed. Complications related to catheter insertion, being an immediate dichotomous event, were evaluated using adjusted ORs, for which the applied statistical model was a multivariable logistic regression, where the dependent variable (dichotomy) was the complication (yes/no), the principal independent variable was the type of insertion (performed by nurse or physician), and other covariables, like relevant clinical characteristics. The model estimated the odds of complication in each group and then calculated:




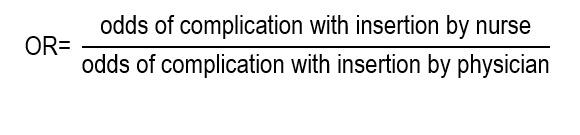




The estimated OR of 0.31 (CI_95%_: 0.17-0.59; *p*<0.001) indicates that the odds of having a complication during or immediately after the PICC insertion was approximately 69% lower when the procedure was carried out by specialist nurses.

 In contrast, the risk of CLABSI, by depending on the time of exposure and the follow-up variable, was analyzed through a Cox proportional hazards model, expressed as HR. The calculation of HR for CLABSI over time is consistent, given that the event can occur during different moments, where not all patients have the same follow-up time, and where some may not present the event (censure). This analysis for each patient considered: i) Time since the PICC insertion until the presence of CLABSI, end of follow-up or catheter removal (censure); ii) as principal variable, the insertion group (nursing vs. physician) was considered. The authors used a Cox proportional hazards model, which estimates the instantaneous hazard:




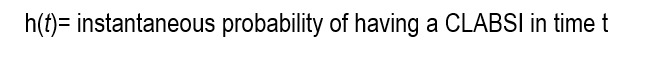




The HR compares those functions between groups:




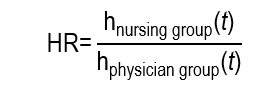




The HR 0.96 (CI_95%_: 0.53-1.75; *p*<0.90), quite close to 1, indicates that, at any moment of the follow up, the instantaneous rate of CLABSI was similar between both groups, without evidence of relevant risk difference over time. It must be kept in mind that an HR close to 1 does not contradict an OR beneficial for another outcome, it simply answers a different question, in a distinct time frame. This example illustrates how the same nursing intervention may require different association measures according to the type of outcome evaluated. 

Impact measures: conceptual foundations and interpretation

Impact measures quantify the absolute effect of an intervention over the clinical outcome, which permit estimating how many events are actually avoided or produced. Among the most relevant there are the risk difference (RD) -also called absolute risk reduction (ARR) when the effect is beneficial-, the absolute risk increase (ARI), the relative risk reduction (RRR), the number needed to treat (NNT), and the number needed to harm (NNH). The RD is an impact measure that expresses the absolute difference in the probability of an event occurring between an exposed or intervened group and an unexposed or reference group. This measure quantifies the excess or absolute risk reduction attributable to an exposure or intervention and can adopt values between −1 and +1 (or between −100% and +100% if expressed in percentages), with the zero (0) value indicating the lack of difference between the groups. Thus, the RD expresses the absolute change in the probability of an event occurring attributable to an intervention, constituting one of most useful measures for clinical decision making. It is calculated as:




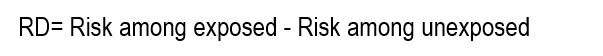




Thereby, RD corresponds to a measure of clinical impact, given that it expresses the absolute change in the probability of an event occurring attributable to an intervention. Thus, while relative measures describe the strength of association, the RD quantifies the absolute impact of an intervention and its clinical relevance. When the outcome is adverse and the intervention reduces it, the RD is expressed as ARR, which corresponds to the direct difference in the probability of the event occurring between the control group and the intervention group. This measure permits estimating how many events are actually avoided by applying a specific intervention:




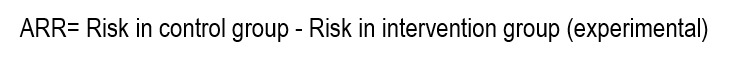




Conversely, when the intervention increases the probability of an adverse event, the RD is expressed as ARI:




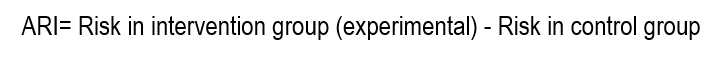




The RRR corresponds to the ratio between the ARR and the risk observed in the control group, and expresses the proportion by which an intervention reduce the risk of an outcome in relative terms. However, by not incorporating the magnitude of the baseline risk or the absolute number of events avoided, the RRR does not allow dimensioning the real clinical impact of the intervention and may lead to overestimating the benefit when interpreted in isolation. Mathematically, it is expressed as:




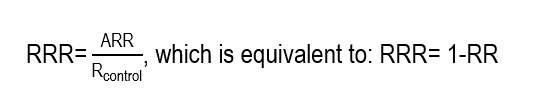




From the ARR, it is possible to calculate the NNT, which is defined as the number of patients that must receive an intervention during a given period to prevent an adverse outcome or produce an additional beneficial result compared to an alternative intervention or an intervention with the control group. Its estimate corresponds to the inverse of the ARR: 




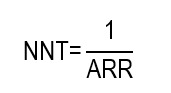




From a conceptual point of view, the minimum theoretical value of the NNT is 1, which would indicate that each patient treated benefits directly from the intervention. A parameter closely related to the NNT is the NNH, which constitutes an absolute impact measure of the harm associated with an intervention. This indicator expresses the number of people who must receive an intervention for an additional adverse event attributable to it to occur, compared with a reference or control group. Its calculation is pertinent when the risk of the harmful outcome is greater in the group undergoing the intervention than in the group receiving the standard treatment or placebo. Hence, the NNH translates a risk difference into a clinically interpretable number that expresses potential harm and must be interpreted together with the NNT to assess the balance between benefits and risks. It is calculated as the inverse of the ARI:




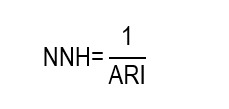




**Chart 3:**
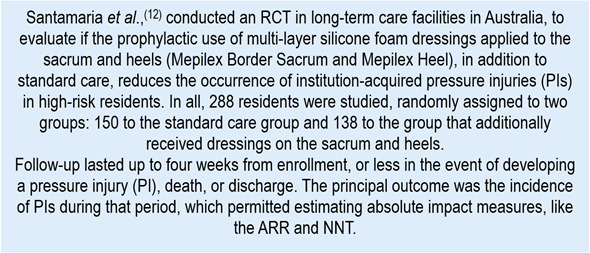
Example of clinical scenario to evaluate RD, ARR, RRR, and NNT

As with the exemplification of risk measures, an article will be used that evaluates a nursing intervention to contextualize the clinical scenario that allows understanding the calculation and interpretation of ARR, RRR, and NNT (Chart 3).

The RCT by Santamaria *et al*.,^12^ reports differences in the principal outcome between the groups studied (PI incidence, that is AR of each group). In the intervention or experimental group (dressings + standard) 3 residents of the 138 developed PIs (incidence = 2.1%), while the control group (standard) registered 16 individuals of the 150 who developed PIs (incidence = 10.6%).




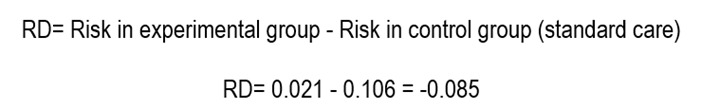




Because the outcome is adverse (PIs), a negative RD implies benefit (-8.5 percentage points). From the same data shown, the clinical equivalent (mostly used) establishes the possibility of calculating the ARR: 




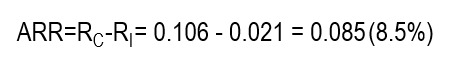




This means that incorporating prophylactic dressings into standard care prevented approximately 9 PIs per every 100 high-risk residents treated during the follow-up period.

Moreover, the authors report the RRR that reached 80%. This fits the following calculation:




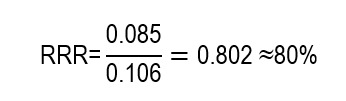




In proportional terms, risk was reduced by approximately 80% with respect to the control. While the RRR is considered high, it does not express how many events are avoided; that is why it is interpreted together with ARR and NNT.

In that sense, the article also exposes the NNT for the intervention in study reported a value of 11.8 (~ 12). This was derived in the following calculation:




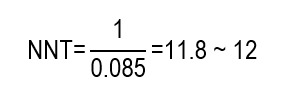




This result implies that prophylactic dressings (in addition to standard care) need to be applied to 12 residents to prevent 1 PI during the follow-up period.

**Chart 4:**
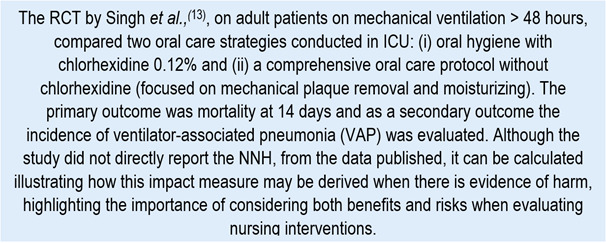
Example of clinical scenario to evaluate NNH

In the clinical trial used to exemplify the impact measures,^12^ the intervention evaluated was associated with a significant reduction in the risk of developing PIs, without evidencing an increase in the occurrence of adverse events attributed to the intervention. Within this context, RD is negative and is expressed as ARR, which permitted calculating impact measures aimed at clinical benefit, such as the NNT. Given that calculating the NNH requires the existence of an ARI in the group intervened, it is not methodologically pertinent to estimate it in this example. In nursing research, the NNH is not often reported in explicit manner in primary studies, even when analyzing interventions widely implemented in the clinical practice. This is due, in part, to many studies prioritizing the evaluation of beneficial outcomes and not incorporating adverse events as primary outcomes. Nevertheless, when studies report incidences of harmful events per group, It is possible to estimate the ARI and derive the NNH, provided that it is explicitly stated that it is a secondary calculation based on the reported data. To exemplify the calculation and use of the NNH, we will analyze the clinical scenario described in Chart 4. 

Despite the study by Singh *et al.,*^13^ not reporting directly the NNH, this can be estimated from the reported AR (incidence). Mortality at 14 days was 67.9% in the group with chlorhexidine (intervention) and 54.5% in the group without chlorhexidine (control), which implies an ARI of 13.4% (0.679 - 0.545 = 0.134). This means that, for every 100 patients, oral care with chlorhexidine is associated, on average, with 13 additional deaths in 14 days, compared with the protocol without chlorhexidine, under the study conditions. Similarly, the incidence of VAP was 40.2% in the experimental group and 23.3% in the control group, corresponding to an ARI of 16.9% (0.402 - 0.233 = 0.169). This is equivalent to approximately 17 additional VAP episodes for every 100 patients exposed to chlorhexidine during 14 days, compared with the protocol without chlorhexidine. From the ARI, it is possible to calculate the NNH as the inverse of said value. For the mortality outcome:




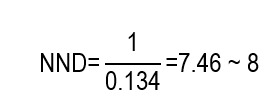




For the VAP outcome, it is obtained that:




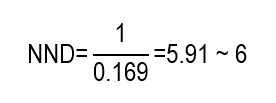




Consequently, the NNH estimated for mortality indicates that, for every 8 patients exposed to chlorhexidine rather than to the protocol without chlorhexidine, on average one additional death occurred during the period evaluated, while the NNH for VAP suggests that exposing approximately 6 patients would be associated with an additional episode of VAP within the same time horizon. 

Risk and impact measures fulfill complementary functions in nursing research. The first describe the relative magnitude of the association between an intervention and an outcome, while the latter permit quantifying the absolute effect and its clinical relevance. Integrated comprehension of both dimensions is essential to interpret adequately the evidence and support informed and patient-focused care decisions. Table 1 summarizes the principal risk and impact measures used in nursing research, integrating their definitions, clinical interpretation, and use. This synthesis seeks to facilitate the critical reading of primary studies and support informed decision making in the clinical practice.

**Table 1:** Risk and impact measures in nursing research: definitions and clinical interpretation

Type of measure	Measure	Definition	Clinical interpretation	Principal use in nursing
Risk measures / association	Relative risk (RR)	Ratio between the outcome risk in the intervened/exposed group and the risk in the unexposed/control group.	How many times greater or less is the risk in the exposed group compared to the non-exposed group.	Cohort studies and clinical trials; frequent in analytical observational research in nursing.
	Odds ratio (OR)	Ratio between the odds of the outcome in the exposed group and in the unexposed group.	Approximates the RR when the event is infrequent.	Case-control studies and multivariate models in nursing observational studies.
	Hazard ratio (HR)	Ratio between the instantaneous rates of the event’s occurrence over time between two groups.	Difference in the rate of the event’s occurrence during the follow up.	Cohort studies and clinical trials with survival analysis in nursing clinical contexts.
Impact measures (absolute effect)	Risk difference (RD)	Absolute difference in the probability of the event occurring between two groups.	Absolute change of the risk attributable to the exposure or intervention.	Clinical translation of the effect in observational and experimental studies.
	Absolute risk reduction (ARR)	RD when the intervention reduces an adverse outcome.	Absolute number of events avoided.	Evaluation of the actual clinical benefit of nursing interventions.
	Absolute risk increase (ARI)	RD when the intervention increases an adverse outcome.	Absolute number of additional adverse events.	Evaluation of the potential harm associated with interventions or exposures.
	Relative risk reduction (RRR)	Proportion by which the intervention reduces the risk with respect to the control group.	Relative magnitude of the effect, independent of the baseline risk.	Complementary measure in the interpretation of experimental and observational studies.
	Number needed to treat (NNT)	Number of patients who must receive an intervention to prevent an additional event.	Clinical efficiency of a beneficial intervention.	Prioritization of preventive and therapeutic interventions in nursing.
	Number needed to harm (NNH)	Number of patients who must be exposed to an intervention to produce an additional adverse event.	Clinical risk associated with an intervention.	Evaluation of the benefit-harm balance in clinical decision making.

## Conclusion

The correct interpretation of risk and impact measures is an essential component of evidence-based nursing practice. This article has systematically addressed the conceptual concepts, calculation, and interpretation of the principal measures used in clinical research, highlighting the complementarity between the relative indicators -which describe the strength of association- and the absolute indicators -which permit dimensioning the real clinical impact of the interventions. Through examples from primary studies in relevant contexts for the nursing practice, it has been illustrated how these measures can be translated into clinically significant information, facilitating informed decision making and analysis of the balance between benefits and risks. Enhancing the methodological skills to select, calculate, and interpret adequately these measures not only improves the critical reading of the scientific literature, but also contributes to a more thoughtful, safe, and contextualized application of evidence in caring for people.
